# COVID-19 messages in sponsored social media posts: The positive impact of influencer-brand fit and prior parasocial interaction

**DOI:** 10.1371/journal.pone.0276143

**Published:** 2022-10-14

**Authors:** Ágnes Buvár, Sára Franciska Szilágyi, Eszter Balogh, Ágnes Zsila

**Affiliations:** 1 Institute of People-Environment Transaction, ELTE Eötvös Loránd University, Budapest, Hungary; 2 Institute of Psychology, ELTE Eötvös Loránd University, Budapest, Hungary; 3 Institute of Psychology, Pázmány Péter Catholic University, Budapest, Hungary; Universitat Autònoma de Barcelona: Universitat Autonoma de Barcelona, SPAIN

## Abstract

This study explores the dissemination potential of a COVID-19 message embedded in a sponsored social media post. The moderating role of prior parasocial interaction and influencer-brand fit were considered. 365 respondents participated in the study. A 3 (control, congruent, incongruent brand) × 2 (control, COVID-19 message) between-subject online experiment was designed and executed. Data were analyzed using a mediated moderation model. Results indicated that the three-way interaction of the COVID-19 message, brand presence and prior parasocial interaction affected the perceived influencer credibility, attitudes towards the social media post, and the behavioral engagement with the post. When the COVID-19 message was included in the post, increased prior parasocial interaction intensified the positive effect of influencer-brand fit on influencer credibility, which in turn resulted in a more positive attitude towards the post, and a higher behavioral engagement. Theoretical and practical implications were discussed.

## Introduction

The COVID-19 outbreak had drastically changed the world and affected our everyday life, including social media use habits. During the pandemic, people have spent more time online than ever [1, 2]. Social media usage increased by 61% over normal usage rates during the first wave of the COVID-19 pandemic [3]. Social media has become a major communication channel in disseminating information, including information related to the virus [4, 5]. Besides, social media platforms are considered adequate channels to disseminate health messages [6, 7].

Social media influencers—content providers exerting social influence on their audience—also started posting about the COVID-19 pandemic, sharing health-related information, writing about their personal experiences or encouraging new behavioral norms such as social distancing and good hygienic practices [8, 9]. Influencers are considered as opinion leaders who can influence their audience’s attitudes and behaviors [10, 11]. Thus, when sharing COVID-19 content, a major role of influencers could be the cultivation of new practices and behavioral norms [8, 12]. Indeed, several articles emphasized the importance of social norms in cultivating COVID-19 preventive behaviors [13–15]. To date, influencers still create and share content about brands as social media provide a source of revenue for them. Consequently, COVID-19 related content is also prevalent in sponsored posts. However, the audience might perceive the presence of a COVID-19 message in a sponsored post as a marketing strategy not as an altruistic advice from the influencer.

Based on previous evidence on the prominent role of influencers in health promotion through social media messages [16], the potential opportunistic perception of COVID-19 messages in brand-related posts [17], and the positive effect of parasocial experience on the persuasive outcomes regarding influencer marketing [18, 19], this study aims to provide a deeper insight into the underlying mechanisms of health communication via sponsored social media messages posted by influencers. Specifically, this study applies an experimental design to investigate the simultaneous moderating effect of prior parasocial interaction and the presence of a COVID-19-related communication in a branded social media post on influencer credibility that in turn affect indirectly the dissemination potential of the social media post.

### Social media influencers and the influencer marketing

Social media influencers acquire followers on social media platforms by creating and sharing content [20, 21]. Besides posting about a specific topic or their everyday life, influencers often take a financial advantage of their popularity by engaging in marketing activities [20, 22]. 69.4% of the influencers stated that their goal is to earn revenue [23]. According to a 2018 survey, 73% of the influencers reported they had posted sponsored content on their feed [24].

Influencers are interested in acquiring new followers, as a larger follower base increases their compensation for a sponsored post [25]. Therefore, influencers attempt to increase their visibility on social media platforms by intentionally using social media algorithms [26]. Taking advantage of these algorithms, social media environments incite and facilitate the discovery of new content by exposing users to previously unseen posts. Consequently, besides studying the reaction of followers, it is also worth examining how a general audience would react to social media influencer posts.

### Influencer-brand fit in sponsored posts

The perceived influencer-brand fit—the perceived connection between the influencer and the advertised brand–plays a crucial role in the sponsored post’s persuasive effect [27]. The match-up hypothesis postulates that an endorsement is more effective when the endorser and the advertised brand are congruent [28].

The effect of influencer-brand fit can be explained by the attribution theory [29] which posits that when there is a close fit between the advertised brand and the endorser, people assume the endorsement reflects the true feelings of the influencer (e.g., the influencer likes the product). However, when there is a perceived incongruence between the brand and the endorser, people are prone to believe that the influencer has ulterior motives (e.g., financial purposes). Consequently, higher consistency between an influencer and the sponsored product can increase the perceived credibility of the endorser [29, 30], which in turn, has a positive impact on persuasion effectiveness [31–33]. A close “match-up” has proved to be effective not only in profit-oriented marketing settings but also in non-profit contexts such as health campaigns [34].

### COVID-19 messages in sponsored posts

With the rise of the COVID-19 pandemic, influencers started posting coronavirus-related content which included posting about safety measures and other information supporting prevention efforts [1, 8, 35]. Thus, influencers faced the challenge of integrating COVID-19-related content in their feed while maintaining income from sponsored posts [12]. A possible solution to the above issue was to include the COVID-19 message directly in the sponsored post.

A recent study suggests that posting about the safety measures and other COVID-19-related information might increase the influencer’s credibility [1]. However, the inclusion of the COVID-19 message in a sponsored post can pose some issues as prosocial messages are often commoditized to serve commercial purposes. Similar to brand opportunism [17, 36], when influencers share a COVID-19 message in a sponsored post, users might attribute self-serving motives to them such as increasing the success of the promotion. On the other hand, users can also attribute altruistic motives to the influencer such as the goodwill towards others [17].

### Parasocial interaction

Parasocial interaction can be defined as a one-way emotional bond with a media figure [37], which is characterized by feelings of being engaged in a real social interaction with the persona (real or fictional character, celebrity, influencer) [38, 39]. A parasocial interaction is based on the viewers’ illusory processes: (1) the illusion of experiencing a face-to-face encounter, resulting in reactions that are generally observed in direct social interactions, and (2) the perception of mutual awareness and the “presence” of the persona that contribute to the sense of reciprocity [40–42].

Previous studies found that individuals showing stronger parasocial interaction perceived the persona/influencer to be more attractive and credible [39, 42–44]. Furthermore, parasocial interaction increases adherence to social norms, and leads to higher level of enjoyment of a media content [39]. In addition, a stronger parasocial experience can lead to more favorable evaluation of sponsored posts, better brand evaluation, stronger purchase intent, and higher willingness to eWOM [18, 19, 43, 45].

When consuming influencer generated content, consumers usually see several posts from the feed of the influencer. Consequently, if the first post evokes positive parasocial experience, it would also affect the reaction to the rest of the content. Since stronger parasocial interaction leads to higher involvement and higher influencer credibility, we posit that a subsequent message from the same source would benefit from these positive effects, and ultimately the message would be evaluated in a more positive way compared to weaker prior parasocial experience. Furthermore, when the prior parasocial experience is stronger, users might evaluate the sponsored post with the COVID-19 message in a more favorable way, attributing an altruistic persuasive motive to the influencer that would result in increased source credibility.

### The moderating effect of the COVID-19 message and prior parasocial interaction on the evaluation of the sponsored social media post

To date, no studies have examined how a COVID-19-specific message included in a sponsored social media post could possibly affect the influencer’s credibility. Besides, no previous studies compared the overall evaluation of a sponsored post involving a COVID-19-specific message using a congruent and an incongruent brand exposure. Based upon the lack of empirical investigations into the nature of these associations, the purpose of this study is to examine the effectiveness of a COVID-19-specific message that encourages compliance with lockdown regulations. This message was embedded in a sponsored social media post created by an influencer who was unfamiliar to the viewers.

**Fig 1:**
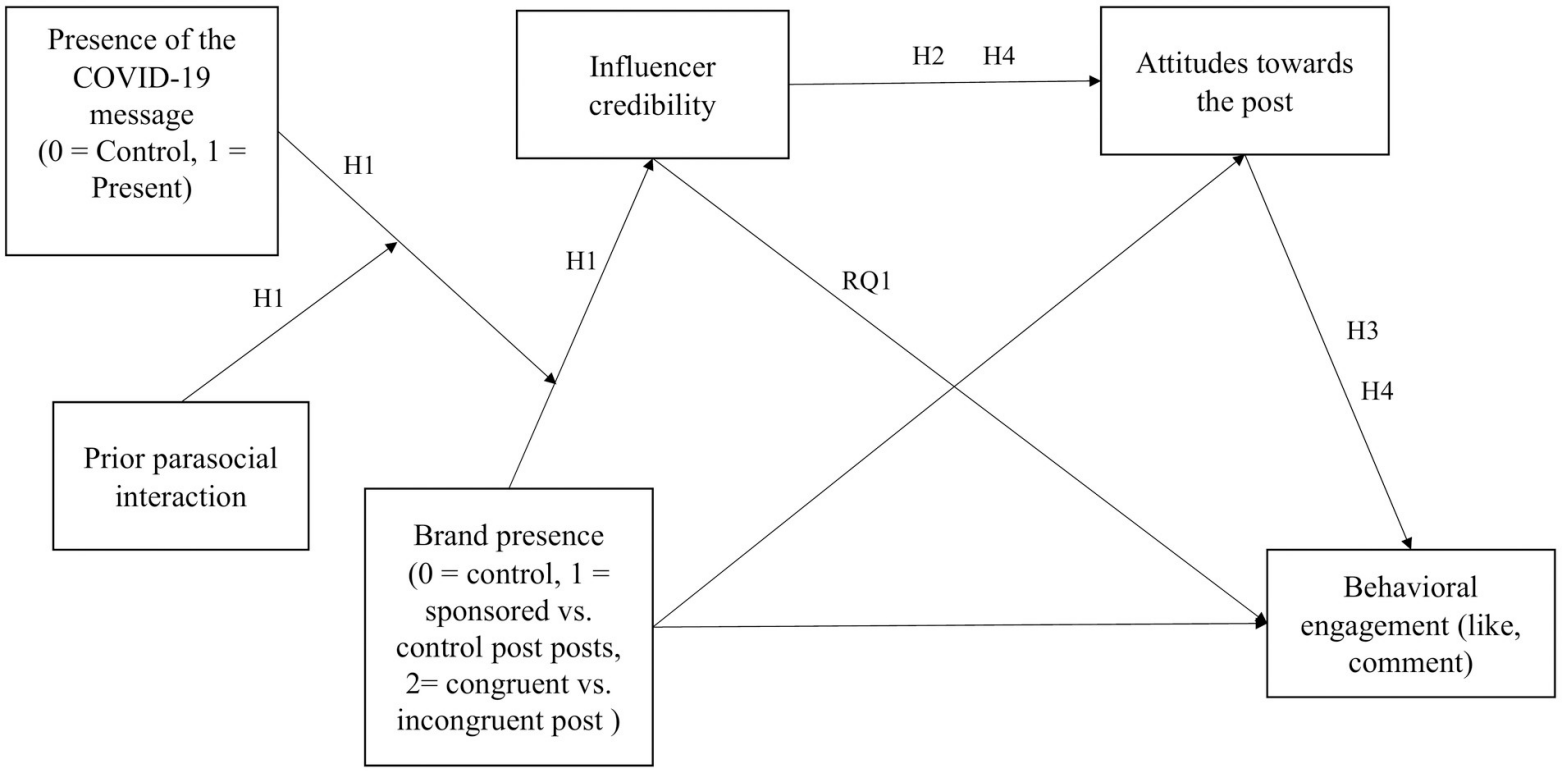
The research model of the study.

Based on the presented literature review, we propose the following hypothesis (see Fig 1):

H1: If the COVID-19 message is included, we posit that when the post is not sponsored (control condition), respondents are more likely to perceive the COVID-19 message as altruistic persuasion. However, when the post is sponsored, it is likely that they would suspect that the COVID-19 message is used as a marketing strategy that would have a negative effect on the credibility of the influencer. Nevertheless, a close influencer-brand fit can lead to higher perceived influencer credibility. Thus, we hypothesize that when the COVID-19 message is included in the post, the congruent brand condition will have a more positive effect on the influencer’s credibility compared to the incongruent brand condition especially when prior parasocial interaction is stronger.

### Influencer credibility and attitudes towards the post

Influencers are perceived as authentic, everyday people who are more accessible and credible than traditional celebrities [46, 47]. Source credibility—as a general trust in the source of a communication [48]–is considered as a crucial factor in the effectiveness of influencer communication [31, 49, 50]. Moreover, previous studies analyzing sponsored posts indicated that influencer credibility is positively related to persuasive outcomes such as attitudes towards the post, attitudes towards the brand or purchase intent [21, 33, 45, 51–53]. Evidence also suggests that source credibility has a prominent role in successful health-related communication including pro-vaccination [54] and tobacco control [55]. Based on this consideration, the second hypothesis is as follows (see Fig 1):

H2: Influencer credibility is associated with more favorable attitudes towards the post.

### Behavioral engagement with social media posts

As previously mentioned, social media can be an adequate platform to promote health messages and raise awareness [56]. Previous results indicated that attitudes towards a social media post can positively influence consumer engagement behavior [57]. Consumer engagement behavior encompasses the users’ reaction to the social media post, and it is linked to the proliferation of the post [57, 58]. Thus, the third hypothesis and the first research question are as follows (see Fig 1):

H3: More positive attitudes towards the post are associated with a stronger behavioral engagement with the post.

RQ1: Besides having an indirect positive effect on behavioral engagement, can influencer credibility directly affect the consumer engagement with the post?

Finally, based upon the hypothetical moderation effect described in H1 and the mediation effect described in H2-H3 along with RQ1, the fourth hypothesis is as follows:

H4: The effect of the three-way interaction of the brand presence, COVID-19 message and prior parasocial interaction on influencer credibility will indirectly affect behavioral engagement through attitudes towards the post.

## Materials and methods

### Study design

We designed a 3 (control, congruent, incongruent brand) × 2 (control, COVID-19 message) between-subject online experiment (see Fig 1). Participants (N = 365) were randomly assigned to six conditions: no brand, no COVID-19 message (n = 67), no brand, COVID-19 message (n = 66), low influencer-brand fit, no COVID-19 message (n = 66), low influencer-brand fit, COVID-19 message (n = 56), high influencer-brand fit, no COVID-19 message (n = 53) and high influencer-brand fit, COVID-19 message (n = 57). Additionally, we measured the third independent variable, prior parasocial interaction. The outcome variable (behavioral engagement) and the two moderator variables (influencer credibility and attitudes towards the post) were also assessed with the appropriate scales and items.

The ethical permission of the current work was issued by the Research Ethics Committee of Eotvos Loránd University, Faculty of Education and Psychology (No 2020/414-3).

### Stimulus material

#### Pretest

A pretest was conducted to select the right stimuli for the experiment. Overall, we tested sixteen different sponsored Instagram posts from four different influencers. Each post from the same influencer contained a different brand. To improve the study’s ecological validity, we selected posts that the influencers previously had published on their Instagram account. Respondents (N = 50) saw all the posts in randomized order, and for each post, they were asked to evaluate the influencer-brand fit. Posts from each influencer were analyzed using repeated-measures ANOVA. Table 1 summarizes the results.

**Table 1:** Results of the pretest.

	Mauchly’s test of Sphericity			One-way repeated measures ANOVA		
	χ^2^	p	Greenhouse-Geisser ε	F	p	η_p_^2^
Influencer 1	χ^2^(5) = 12.0	.035	.868	F (2.604, 147) = 47.5	< .001	.492
Influencer 2	χ^2^(5) = 19.3	.002	.806	F (2.419, 147) = 70.4	< .001	.590
Influencer 3	χ^2^(5) = 8.58	.127	.910	F (3, 147) = 44.4	< .001	.475
Influencer 4	χ^2^(5) = 9.50	.091	.887	F (2.661, 147) = 22.8	< .001	.317

Results indicated that the tested posts significantly differed regarding the influencer-brand fit for all four influencers. Final decision was to use Influencer 4, a female lifestyle influencer having 431 000 followers. She is relatively famous among adolescents, but less known among the 18+ population (good fit post: M = 5.55, SD = 1.23 versus poor fit post: M = 3.55, SD = 1.69, paired samples t-test: t(49) = 7.74, p < .001).

In the high influencer-brand fit condition, the influencer advertised a Coco Wow facial mask from the brand Hello Body with a photo taken in a bathroom (the original post is available from: https://www.instagram.com/p/B66NJPmp0SH/). In the low influencer-brand fit condition, she promoted a pizza brand while playing a videogame (FIFA 20) (the original post is available from: https://www.instagram.com/p/CGmkzCsJmdE/). We also chose a control (not sponsored) post where she is posing on the bed (the original post is available from: https://www.instagram.com/p/CF1nfK-Jpbh/). The text was modified to insert the COVID-19 message in the post. We included the same message in each post: *“Lately*, *because of the lockdown*, *I have much spare time*, *because I cannot go anywhere… You should also stay at home and take care*!*”*. We also included *#covid* at the end of the post.

### Participants

Overall, 370 Hungarian respondents participated in the study. Five respondents were eliminated because they indicated that they were less than 18-year-old. The final convenience sample consisted of 365 respondents (10 missing data, M_age_ = 29.9, SD_age_ = 10.9, Min_age_ = 18, Max_age_ = 72; 71.6% female). 49.4% of the respondents owned at least a BA degree, while an additional 28.3% had some college credits without any degree (4 missing data). 84.1% claimed to have no or basic education in marketing and advertising (4 missing data).

### Procedure

Students who fulfilled course requirements recruited the respondents by sharing the link of the experiment on social media platforms where they were present, mainly on Facebook, Instagram and YouTube. Data was collected between December 2020 and May 2021 using a website programmed by one of the authors. Anyone over 18 years of age could participate in the study. After consented to participate in the study, respondents were asked whether they were familiar with the selected influencer. If they responded no, they viewed a 2-minute video about her that compiled several videos edited by one of the authors. Respondents could not move on with the questionnaire before the video was finished. Then, parasocial interaction was assessed. Next, respondents were randomly exposed to one of the six Instagram posts for a minimum of ten seconds. Respondents were allocated to conditions using simple randomization. The post was followed by questions about the post, the brand and the COVID-19 message depending on which post they saw. Then, source credibility was assessed. We included an instruction manipulation check *(“To answer this question*, *please select the fourth button from the left*.*”*) among source credibility questions to check the respondents’ attention. Those who did not answer correctly could not finish the questionnaire; their previous answers were immediately deleted. Finally, demographic questions were asked. At the end of the questionnaire, respondents were debriefed.

### Measures

The scales and items were translated from English using the Beaton-Bombardier process regarding the cross-cultural adaptation of self-report measures [59].

#### Parasocial interaction

The Experience of Parasocial Interaction Scale consisted of six items [39], for instance: “While watching the clip, I had the feeling that [the influencer]…“was aware of me” or “knew I was aware of him/her”. Answers were given on a seven-point Likert scale ranging from 1 = “not at all agree” to 7 = “definitely agree”. The items indicated excellent reliability; therefore, items were averaged to create a single variable (Cronbach α (6 items) = .875, M = 2.06, SD = 1.18).

#### Source credibility

The Celebrity Endorser Credibility Scale consisted of three dimensions: expertise, trustworthiness, and attractiveness [48]. Five items measured each dimension such as “expert”–“not an expert” or “qualified”–“unqualified” (expertise), “honest”–“dishonest”, “reliable”–“unreliable” (trustworthiness) and “attractive”–“unattractive”, “elegant”–“plain” (attractiveness). Answers were given on a five-point semantic differential scale. A summed variable including all dimensions was created for further analysis (Cronbach α (15 items) = .919, M = 35.1, SD = 10.4, Min = 15, Max = 70).

#### Influencer-brand fit

The influencer-brand fit was assessed using the following question: “How would you describe the relationship between the [brand] and the [influencer]?”. Answers were given on a 7-point semantic differential scale using the following items: “inappropriate”–“appropriate”, “inconsistent”–“consistent”, “unlikely match”–“likely match”, “irrelevant”–“relevant” [32]. Items were averaged to create a single variable (Cronbach α (4 items) = .903, M = 4.33, SD = 1.56).

#### Attitudes towards the post

Attitudes towards the post and the COVID-19 message were assessed using a five-item semantic differential scale: “dislike”–“like”, “negative”–“positive”, “boring”–“interesting”, “unappealing”–“appealing”, “annoying”–“not annoying”. Answers were given on a 7-point scale. Items were averaged to create a single variable for both measures (Cronbach α (5 items) = .879, M = 2.56, SD = 1.25).

#### Behavioral engagement with the post

To assess behavioral engagement with the post, respondents answered the following question: "To what extent do you think it is likely that due to the post, you would perform the following activities?”. We assessed two activities: liking and commenting [57]. Answers were given on a 7-point scale ranging from “Definitely unlikely” to “Definitely likely”. Given the acceptable reliability, we averaged the items to create a single measure of behavioral engagement (Cronbach α (2 items) = .647, M = 1.31, SD = 0.749). Due to the non-normal distribution of the variable (skewness = 3.04, kurtosis = 9.96), we applied a logarithmic transformation that we used in the subsequent analyses.

#### Presence of COVID-19 message in the post

We asked those who viewed a post with the COVID-19 message what messages they recall from the post (open-ended question). Each respondent could describe a maximum of five messages.

### Data analysis

First, descriptive analyses of the predictor, moderator, mediator and outcome variables were conducted. Then, we performed a manipulation check whether the influencer-brand fit was perceived as intended. We also verified the distribution of the demographic and control variables among the experimental groups.

The main analyses consisted of conducting a moderated mediation analysis using ordinary least squares regression. The model examined the indirect effect of brand presence, prior parasocial interaction and the presence of the COVID-19 message on the behavioral engagement with the post mediated by the attitudes towards the post and the influencer’s credibility. All continuous variables were standardized before the analysis. The statistical analyses were conducted using SPSS Statistics v.26; the moderation analyses were performed using a modified version of model 83 of PROCESS macro v3.4.1 [60].

## Results

### Manipulation check and distribution of demographic variables across experimental groups

We conducted a t-test to assess whether the intended manipulation of the influencer-brand fit was successful. Results indicated that in the high influencer-brand fit condition, the brand was perceived as more congruent compared to the low fit condition (M_low_ = 4.13, SD_low_ = 1.59 versus M_high_ = 4.54, SD_high_ = 1.49; t(230) = -1.99, p = .047, d = -.262).

Among the 179 respondents who saw a post with a COVID-19 message, 68.2% gave at least one valid answer to the open-ended question about the messages they can recall from the post they had seen. Among those who gave a valid answer (122 respondents), 63.1% mentioned at least one COVID-19 related message.

We conducted Chi-square tests to assess the equal distribution of gender, education, and marketing expertise across experimental groups. Results indicated equal distribution across experimental groups (all ps < .087). Thus, gender, education, and marketing expertise did not explain the effect of the manipulated variables.

Finally, we conducted one-way ANOVAs to assess the equal distribution of age and familiarity with social media marketing across the experimental groups. Results indicated that the distribution was equal across experimental groups (all ps < .583). Thus, age and familiarity with social media marketing did not explain the effect of the manipulated variables.

### Mediated moderation analysis

We conducted a mediated moderation analysis with the brand presence as the predictor variable, the presence of the COVID-19 message, and the prior parasocial interaction as moderator variables, the source credibility and the attitudes towards the post as mediator variables and behavioral engagement as the outcome variable. We applied Helmert contrast coding for the brand presence variable: first, the branded posts were compared to the control post, then the two branded posts containing the congruent and the incongruent brand were compared, respectively. We used a modified version of model 83 of the PROCESS macro; confidence intervals were calculated using 5 000 bootstrapped samples and heteroscedasticity-consistent standard errors (HC3).

**Fig 2:**
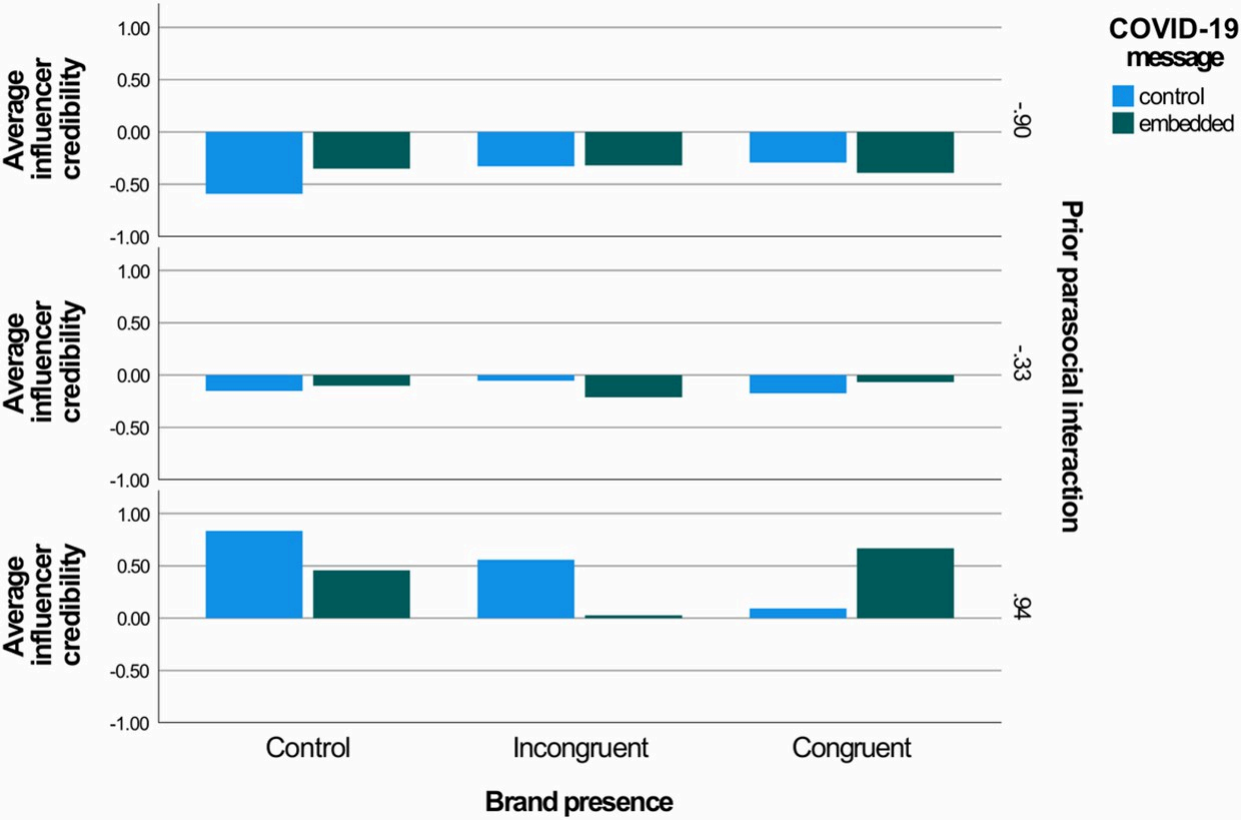
The conditional effect of the brand presence-COVID-19 message interaction at different strengths (weak, moderate, strong) of prior parasocial interaction.

Regarding the first hypothesis (H1) about the moderating effect of the COVID-19 message and prior parasocial interaction on the effect of brand presence on influencer credibility, results indicated a significant three-way interaction (R^2^ change = .026, F(2, 353) = 4.75, p = .009). The brand presence, prior parasocial interaction and the presence of the COVID-19 message simultaneously affected the influencer’s credibility (see also Fig 2).

First, results indicated that the conditional interaction of the brand presence and the COVID-19 message was only significant when prior parasocial interaction was strong (at the 84^th^ percentile) (F(2, 353) = 5.99, p = .003). When prior parasocial interaction was weak (at the 16^th^ percentile) or moderate, neither the presence of the brand, nor the COVID-19 message affected influencer credibility (all ps are > .116).

On one hand, when prior parasocial interaction was strong and the COVID-19 message was not present, the influencer was perceived as less credible when the post was sponsored compared to the control post (b = -0.508, t(353) = -2.20, p = .028, 95% bootstrapped CI = [-0.961, -0.055). However, the incongruent post led to higher influencer credibility compared to the congruent one (b = -0.464, t(353) = -2.08, p = .038, 95% bootstrapped CI = [-0.902, -0.026]).

On the other hand, when prior parasocial interaction was strong and the COVID-19 message was present, we found no difference between the sponsored posts and the control post regarding influencer credibility (p = .654). Nonetheless, the congruent post led to higher influencer credibility compared to the incongruent one (b = 0.640, t(353) = 2.64, p = .009, 95% bootstrapped CI = [1.65, 1.12). Thus, H1 is supported.

**Fig 3:**
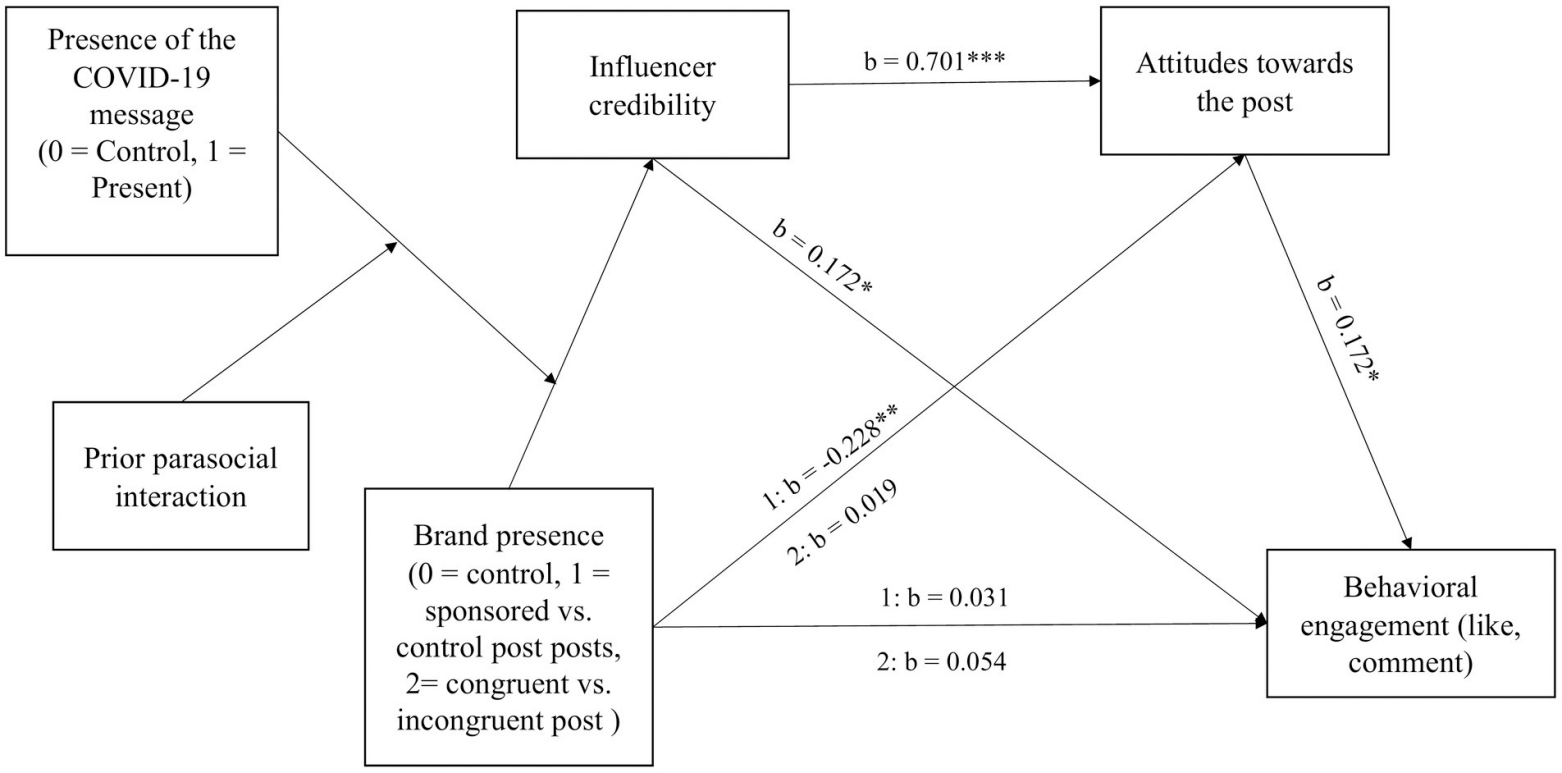
The mediation effects of the research model. *: p < .05, **: p < .01, *** p < .001.

Fig 3 summarizes the mediation effects of the research model. Supporting H2, results indicated that influencer credibility was positively related to the attitudes towards the post (b = 0.701, t(361) = 19.4, p < .001, 95% bootstrapped CI = [0.630, 0.772]).

Regarding RQ1 whether influencer credibility has a direct effect on behavioral engagement besides the indirect effect via the attitudes towards the post, results indicated that influencer credibility also had a direct positive link to behavioral engagement (b = 0.172, t(360) = 2.38, p = .018, 95% bootstrapped CI = [0.030, 0.315]).

Finally, confirming H3, results indicated that attitudes towards the post were also positively related to behavioral engagement (b = 0.306, t(360) = 3.98, p < .001, 95% bootstrapped CI = [0.155, 0.457]).

Subsequently, to test H4 whether the effect of the three-way interaction of brand presence, COVID-19 message presence and prior parasocial interaction affected behavioral engagement indirectly, we examined the moderated mediation indexes of the model. Results indicated that the moderated mediation index was significant when the congruent post was compared to the incongruent one regarding these two paths: influencer credibility → behavioral engagement (index = 0.114, bootstrapped CI = [0.013, 0.252]), and influencer credibility → attitudes towards the post → behavioral engagement (index = 0.142, bootstrapped CI = [0.034, 0.271]). Thus, when prior parasocial interaction was strong, the incongruent post without the COVID-19 message led to better influencer credibility, more positive attitudes and higher behavioral engagement compared to the congruent one; on the other hand, when the COVID-19 message was included, the congruent post led to higher influencer credibility, more positive attitudes and higher behavioral engagement compared to the incongruent one.

Additionally, we found that the brand presence had a negative direct effect on attitudes towards the post: the attitudes towards the sponsored posts were more negative compared to the control post (b = -0.228, t(361) = -2.98, p = .003, 95% bootstrapped CI = [-0.379, -0.078]).

## Discussion

Instagram has become an important source of information, especially among the 18–29-year-old [61]. Prosocial messages such as COVID-19 messages posted by social media influencers can quickly spread and reach a large audience. In this study, we investigated the dissemination potential of a social media influencer’s Instagram post considering different boundary conditions such as prior parasocial interaction, the presence of a congruent/incongruent brand and the presence of a COVID-19 message. We hypothesized that the interaction among the above-mentioned components would affect influencer credibility (H1), which in turn influences the attitudes towards the post, and lead to a stronger behavioral engagement with the post (H2-H4, RQ1).

Regarding our first hypothesis (H1), our results indicated a significant three-way interaction among brand presence, the presence of a COVID-19 message and prior parasocial interaction. The conditional interaction effect between the brand presence and the COVID-19 message on the influencer credibility was significant only when prior parasocial interaction was elevated. Previous studies examining the moderating role of involvement regarding the effect of endorser-brand fit on persuasion outcomes found that the endorser-brand fit only had a positive effect when involvement was high [62–64]. Our findings are in line with previous results, as parasocial interaction can be interpreted as a measure of the viewer’s involvement with the influencer [37]. Thus, prior parasocial interaction could have increased positive attitudes towards the subsequent message. This finding is important, because single social media posts are often studied out of context, while our results prove that the reaction to previous posts in social media feeds could influence how users react to the subsequent message even if the content of the two posts is unrelated.

When prior parasocial interaction was strong and the COVID-19 message was not included, the control post led to higher influencer credibility compared to the sponsored posts. The perception that influencers promote too many brands can explain this result; thus, influencers lose their credibility, weakening the impact of sponsored posts [1, 65]. Furthermore, when the COVID-19 message was not included in the post, the congruent post led to lower credibility than the incongruent one. Although most studies support that the influencer-brand fit leads to better persuasion effects, including the perception of influencer credibility [43, 44], other studies found that less congruent brands or moderate congruency can lead to better evaluations [64, 66]. In this case, even the incongruent influencer-brand fit was perceived more favorably than the arithmetic mean of the measurement scale. Thus, it is more accurate to talk about a less congruent and a more congruent brand. Moreover, the two sponsored posts were also different regarding other characteristics such as the explicitness of sponsorship or the narrative of the text that could have also affected the evaluation of the influencer’s credibility. We chose to use publicly available posts from a real influencer to increase ecological validity; however, a more structured stimuli set enables to control for these differences.

When prior parasocial interaction was strong, and the COVID-19 message was included in the post, we found no significant difference between the sponsored posts and the control. However, the more congruent post led to significantly higher influencer credibility than the less congruent post. This finding is supported by previous results indicating that the influencer-brand fit leads to higher perceived source credibility [43, 44]. Moreover, our results suggest that this positive effect of influencer-brand fit affected the entire post including the COVID-19 message. Alternatively, drawing on the attribution theory, users might have perceived that the influencer genuinely likes the congruent brand, so they also perceived that there was no opportunistic motive behind the COVID-19 message, either. Thus, together these perceptions led to higher perceived influencer credibility. On the other hand, when the advertised brand was incongruent with the influencer, users might have perceived an exterior motive behind the promotion that was exaggerated by the COVID-19 message where they could suspect opportunism. Consequently, users perceived the influencer less credible. The COVID-19 message increased influencer credibility only in the congruent post, while it decreased the credibility in the case of the less congruent brand. It is possible that, as Francisco et al. [1] posited, the COVID-19 message increased the influencer’s credibility, but only when the influencer-brand fit also pointed towards higher influencer credibility.

Regarding the direct effects on the attitudes towards the post, results indicated that the brand presence negatively affected the attitudes towards the post, regardless of the influencer-brand fit. Again, the increasing distrust in sponsored posts due to the proliferation of sponsored messages in social media provides a suitable explanation [1, 65].

Regarding H2 and H3, in line with previous results, our results indicated that influencer credibility was positively related to attitudes towards the post, and attitudes towards the post were positively associated to behavioral engagement. Besides, we found that influencer credibility also has a positive direct effect on behavioral engagement (RQ1).

Regarding H4, our results indicated a serial mediation effect of the above-described three-way interaction that affected source credibility, which in turn was positively related to both the attitudes towards the post and the behavioral engagement with the post. Furthermore, attitudes towards the post were also linked to behavioral engagement that could indicate the proliferation potential of the post (i.e., how fast the post would be shared in the social media) [57]. Consequently, when prior parasocial interaction was strong, the congruent post with the COVID-19 message led to more positive attitudes and higher behavioral engagement, which could suggest a higher proliferation potential of the COVID-19 message.

### Theoretical and practical implications

This study has a number of theoretical contributions to present knowledge on influencer communications. First, in line with the metanalysis of Tukachinsky et al. [67], our results indicated a similarity between prior parasocial interaction and parasocial relationship regarding persuasion outcomes: stronger prior parasocial interaction as well as stronger parasocial relationship led to better persuasion outcomes including behavioral engagement. Furthermore, our results suggest that a stronger prior parasocial interaction leads to higher involvement, while parasocial relationship represents a more complex and intimate relationship. Thus, it is possible that while both parasocial interaction and parasocial relationship have a positive effect on persuasion outcomes, the underlying mechanisms might be different.

In a similar vein, our results provide empirical evidence that prior parasocial interaction with an influencer can affect the evaluation of a subsequent message even in the case of an unrelated content. This area of research requires further exploration as social media posts are often studied out of their original context (the social media influencer’s feed). However, users usually view several posts from the same influencer. As the parasocial relationship develops after repeated encounter with the persona, prior parasocial interaction could be interpreted as the result of the first encounter when the viewer had been previously unfamiliar with the persona. From this perspective, prior parasocial interaction can be interpreted as a weaker version of a parasocial relationship.

Third, we found that when prior parasocial interaction is strong, it enhances the positive effect of the congruent brand on influencer credibility only when the COVID-19 message was present. As explained earlier, attribution theory might provide an explanation for this result. However, the inclusion of the COVID-19 message altered the interaction effect between prior parasocial interaction and the brand-influencer fit. Future research might investigate whether additional elements affect the interaction between (prior) parasocial experience and brand-influencer fit. A strong parasocial interaction might enhance the positive effect of a congruent brand as the present results indicated, but it is also possible that a strong parasocial experience overwrites the effect of congruence as previous studies found [51].

This study provides a more nuanced picture of how a COVID-19 message in a sponsored post can be promoted effectively by influencers. First the results indicated that neither the brand presence, nor the COVID-19 message influenced persuasion outcomes without a strong prior parasocial interaction. Thus, when designing persuasive communication, besides the social media post carrying the persuasive message, content providers should consider the context of the social media post as well. If the influencer’s feed does not sufficiently involve users, they might overlook posts containing prosocial or health messages.

Second, we would recommend not to include a COVID-19 message in a sponsored communication, as the brand presence can negatively affect the overall evaluation of the message, as was demonstrated in this study. However, these negative effects can be mitigated if the sponsored post promotes a brand that is congruent with the influencer. Consequently, including prosocial or health messages in congruent sponsored posts might represent a viable alternative for influencers to reconcile financial interest and noncommercial communication goals within the same social media post.

### Limitations and further research

This study is not without limitations. Although we found a significant difference between the congruent and incongruent brands, as mentioned before, it is more precise to say that one brand was more congruent than the other. Besides, a more structured stimuli set might be used in further research to control for post characteristics such as the posture of the influencer, the narrative of the text or the explicitness of sponsorship. Moreover, we conducted this study using Instagram posts. Instagram is a highly visual platform. Thus, the generalizability of the results might be limited when social media posts are less visual in nature and more textual (e.g., Twitter posts).

We conducted the study using a convenience sample of relatively young participants, and individual differences that we did not control for might have affected the results. For instance, despite the random assignment of the respondents into control and experimental groups, there is a chance that participants differed in prior knowledge/experience about the COVID-19 across various experimental groups. If prior COVID-19 knowledge/experience leads to positive/negative attitudes towards lockdown restrictions, it could presumably affect the mediator and outcome variables of the study. Moreover, prior knowledge/experience related to the COVID-19 virus could also affect the respondents’ involvement with the post and its perceived relevance, which in turn could influence the processing of the post. This mechanism could also affect the study`s results [68]. Further research should consider controlling for prior knowledge concerning the prosocial message that is used. Furthermore, both the respondents’ and the influencer’s gender might affect the strength of parasocial interaction between the respondents and the influencer. For instance, women tend to report stronger parasocial experiences, and there is evidence that they are more open to cross-sex parasocial relationships than men [67]. Moreover, another study found that the celebrity’s perceived attractiveness affects the quality of the parasocial interaction via perceived homophily (similarities between audiences and influencers) [69]. Regarding this study, gender might have affected the perceived attractiveness of the influencer that could have indirectly affected the parasocial interaction. This effect should be investigated in subsequent studies.

Further research can take several directions. Studies can replicate our study, replacing parasocial interaction with parasocial relationship. The role of perceived authenticity could be investigated for both parasocial interaction and parasocial relationship, and their connection to the influencer-brand fit. Video stimuli containing a COVID-19 message could enable the simultaneous assessment of the parasocial interaction and the COVID-19 message efficacy. Other dimensions such as the influencer-cause fit, the brand-cause fit, or the design and the wording of the social media post could also be included in further studies.

## Conclusion

The present findings can increase knowledge of how prior parasocial interaction might affect persuasive communication. Indeed, findings of this study indicate that prior parasocial interaction might be associated with higher levels of involvement that leads to more favorable persuasion outcomes. Moreover, the present results suggest that a strong prior parasocial interaction can possibly intensify the positive effect of influencer-brand congruence on perceived influencer credibility mitigating the risk of including COVID-19 content in a sponsored media communication. Overall, the present findings can contribute to a better understanding of how a COVID-19-specific social media message can affect an influencer’s perceived credibility under different conditions.
